# Socioeconomic Status and Self-Rated Oral Health; Diminished Return among Hispanic Whites

**DOI:** 10.3390/dj6020011

**Published:** 2018-04-24

**Authors:** Shervin Assari

**Affiliations:** 1 Center for Research on Ethnicity, Culture, and Health (CRECH), School of Public Health, University of Michigan, Ann Arbor 48109-2700, MI, USA; assari@umich.edu; 2 Department of Psychiatry, University of Michigan, Ann Arbor 48109-2700, MI, USA 4250 Plymouth Rd., Ann Arbor, MI 48109-2700, USA

**Keywords:** economic inequalities, ethnic health disparities, socioeconomic status, oral health

## Abstract

*Background.* An extensive body of knowledge has documented weaker health effects of socio-economic status (SES) for Blacks compared to Whites, a phenomenon also known as Blacks’ diminished return. It is, however, unknown whether the same diminished return also holds for other ethnic minorities such as Hispanics or not. *Aim.* Using a nationally representative sample, the current study aimed to compare Non-Hispanic and Hispanic Whites for the effects of SES on self-rated oral health. *Methods.* For the current cross-sectional study, we used data from the Collaborative Psychiatric Epidemiology Surveys (CPES), 2001–2003. With a nationally representative sampling, CPES included 11,207 adults who were either non-Hispanic Whites (*n* = 7587) or Hispanic Whites (*n* = 3620. The dependent variable was self-rated oral health, treated as dichotomous measure. Independent variables were education, income, employment, and marital status. Ethnicity was the focal moderator. Age and gender were covariates. Logistic regressions were used for data analysis. *Results.* Education, income, employment, and marital status were associated with oral health in the pooled sample. Although education, income, employment, and marital status were associated with oral health in non-Hispanic Whites, none of these associations were found for Hispanic Whites. *Conclusion.* In a similar pattern to Blacks’ diminished return, differential gain of SES indicators exists between Hispanic and non-Hispanic Whites, with a disadvantage for Hispanic Whites. Diminished return of SES should be regarded as a systemically neglected contributing mechanism behind ethnic oral health disparities in the United States. Replication of Blacks’ diminished return for Hispanics suggests that these processes are not specific to ethnic minority groups, and non-White groups gain less because they are not enjoying the privilege and advantage of Whites.

## 1. Introduction

Ethnic disparities in oral health have been well-established in several studies, with Hispanics and Blacks being at higher risk of poor oral health [1,2]. Hispanics generally have poorer oral health compared to non-Hispanic Whites in the United States. For instance, Hispanic Whites aged 35–44 years are twice as likely to have an untreated tooth decay compared to non-Hispanic Whites, which is mainly due to lower access of minority and low socioeconomic status (SES) individuals to dental care [1]. A major explanation for ethnic disparities in oral health is lower SES status of ethnic minority populations [2,3,4,5,6,7,8,9].

Socioeconomic status indicators such as education attainment and income are protective factors against poor oral health and enhances population access to oral health care services [10,11,12]. Among adults aged 35–44 years, having less than a high school education is associated with three-fold increase in untreated tooth decay in comparison to the individuals having some college education. Individuals with less than a high school education are three times more likely to have destructive periodontal (gum) disease compared to individuals with some college education [1]. Various barriers reduce dental health care use of low educated and low-income individuals including low levels of oral health literacy, not seeing oral health as a major component of overall health, and lower access to oral health care services [2].

Income inequalities are probably the largest contributors to oral health disparities [13]. Low-income reduces the chance of having seen a dental provider over the past year [14]. In 2010, annual dental visits were 42% and 70% in individuals above and below 200% of the federal poverty line [14]. One fifth of low-income adults report no dental visit in the past five years [14]. Several barriers including insurance, transportation, and time flexibility hinder low income individuals from chance of having a dental visit [2].

Unequal gain of equal resources is a systemically neglected cause of health disparities [15]. Different from other explanations such as poor access to the healthcare system [16], low SES [17], high stress [18], and discrimination in the community [19] and health care system [16], minorities diminished return argues that some of the ethnic health disparities are not because of lower SES of ethnic minorities, but lower health gain that follows the very same SES indicators such as education, income, employment, and marital status [15,20].

### Aim

Using a nationally representative sample of non-Hispanic and Hispanic Whites, the current study aimed to explore ethnic variation in the effects of SES on self-rated oral health. In line with the Blacks’ diminished return theory, we expected education, income, employment, and marital status to be associated with oral health in overall sample. We, however, expected stronger protective effects of SES indicators against poor oral health for non-Hispanic and Hispanic Whites.

## 2. Methods

### 2.1. Design and Setting

With a cross-sectional design, the current study used data from the Collaborative Psychiatric Epidemiology Surveys (CPES), 2001–2003. The CPES was conducted by the University of Michigan (UM). Although the CPES methods has been described in detail elsewhere [21], we briefly summarize the study methodology here.

CPES is composed of three national surveys: (1) the National Comorbidity Survey-Replication (NCS-R) [22]; (2) the National Latino and Asian American Study (NLAAS) [23]; and (3) the National Study of American Life (NSAL) [21]. The CPES data were collected by the University of Michigan (UM) Institute for Social Research (ISR), Ann Arbor.

### 2.2. Sampling

Non-Hispanic White and Hispanic White participants were recruited using the CPES core sampling. Core sampling of the CPES was a multistage stratified area probability sample, that recruited a nationally representative household sample. All participants were adults (18 years of age and older). Participants were recruited from households in the coterminous 48 states. The sample was limited to individuals who were able to conduct an interview in English. This study did not include any institutionalized individual. Thus, being in prisons, jails, nursing homes, and medical facilities were exclusion criteria [21]. Non-Hispanic White and Hispanic White in the CPES were selected from large cities, other urban areas, or rural areas [21]. The analysis for the current study included a total of 11,207 adults who were either non-Hispanic Whites (*n* = 7587) or Hispanic Whites (*n* = 3620).

### 2.3. Ethics

The CPES study protocol was approved by the University of Michigan (UM) Institutional Review Board (IRB # B03-00004038-R1). Informed written consent was received from all the participants. Data were kept anonymous. Participants were financially compensated for their time. Publicly available CPES data were downloaded from Interuniversity Consortium for Political and Social Research (ICPSR https://www.icpsr.umich.edu), located at University of Michigan (UM) Institute for Social Research (ISR).

### 2.4. Data Collection

CPES collected data using structured interviews (survey questionnaires). Most of the data were collected using computer-assisted face-to-face interviews. Telephone interviews were only used for the remaining of the data collection. Interviews lasted between 2 and two hours on average. The overall response rate of the CPES is 69%.

### 2.5. Measures

#### 2.5.1. Independent Variables

Household income was self-reported. Income was treated as a continuous measure in this study. To increase interpretability of the income coefficients, we divided income by USD 10,000. So, our income coefficients reflect the effect of a USD 10,000 increase in income on odds of poor self-rated oral health. The socioeconomic covariate included education, which was measured as an ordinal variable with the following four categories: (1) less than 11 years; (2) 12 years; (3) between 13 and 15 years; and (4) 16 years or more. Education was operationalized as a continuous variable.

#### 2.5.2. Dependent Variable

*Self-rated Oral Health.* A single item measure was used for oral health. The item was “How would you rate the overall condition of your teeth, gums, and mouth at the present time?” Responses were (1) Excellent; (2) Very good; (3) Good; (4) Fair; and (5) Poor. The findings suggest that perceived oral health may be a useful outcome measure in dentistry because of its relation to predisposing socio-demographics and dental utilization [24]. Regardless of ethnicity, self-rated oral health reflects oral symptoms [24]. We considered fair/poor oral health as the outcome, with other responses (excellent, very good, good) as the reference group [25].

#### 2.5.3. Covariates

Covariates in this study included demographic characteristics (age and gender). Age was operationalized as a continuous variable. Gender was conceptualized as a dichotomous variable (male 1 vs. female 0).

#### 2.5.4. Moderator

*Ethnicity.* Ethnicity was self-identified in the CPSE [26,27,28,29,30,31,32]. Hispanic Whites were defined as persons who were of Latino, Hispanic or Spanish origin and were not Blacks. Ethnicity was treated as a dichotomous variable, with non-Hispanic Whites being the reference category. (Hispanic Whites = 1 vs. non-Hispanic Whites = 0). Hispanic Whites were composed of from four distinct ethnic subgroups: 868 Mexican, 577 Cuban, 495 Puerto Ricans and 614 Other Latinos.

### 2.6. Statistical Analysis

#### 2.6.1. Weights

To accommodate the CPES’s sampling weight due to the multi-stage sampling design, we used Stata 13.0 (Stata Corp., College Station, TX, USA) for all our data analysis. This approach will generate nationally representative statistics. Taylor series linearization was used to estimate designed based standard errors and variances. To perform our subsample analyses, we applied sub-pop survey commands in Stata.

#### 2.6.2. Analytical Plan

For descriptive purposes, we used mean (SE) and proportions (relative frequency). Bivariate analyses included independent sample *t* test, Pearson Chi square, and Spearman correlation tests in the pooled sample and by ethnicity. For multivariable analysis, we used logistic regression models. Adjusted odds ratios (OR) and *p* values were reported. In our logistic regression models, SES indicators were the independent variables, poor self-rated oral health was the dependent variable, and demographics were covariates. Ethnicity was the focal moderator. The first series of logistic regression models (*Model 1*) estimated the main effects of ethnicity and SES indicators in the pooled sample. Next models (*Model 2*) included one ethnicity by SES interaction term. Then we ran subsequent models in non-Hispanic Whites (*Model 3*) and Hispanic Whites (*Model 4*).

## 3. Results

### 3.1. Descriptive Statistics

Table 1 describes the descriptive statistics for the pooled sample as well as non-Hispanic and Hispanic Whites. Gender was not different in Non-Hispanic and Hispanic Whites. Non-Hispanic Whites were considerably older than Hispanic Whites. Compared to non-Hispanic Whites, Hispanic Whites had lower educational attainment and income. Hispanic Whites were less likely to be married and employed than non-Hispanic Whites.

### 3.2. Bivariate Correlations

Table 2 presents the results of bivariate correlations in the pooled sample. SES indicators including education, income, marital status and employment were correlated with self-rated oral health in the pooled sample (Table 2).

### 3.3. Logistic Regressions in the Pooled Sample

Table 3 presents the results of two series of logistic regression models in the pooled sample with oral health as the outcome. *Model 1* only included the main effects of ethnicity and SES. *Model 2* also included an interaction term between ethnicity and a SES indicator. Based on *Model 1*, education, income, employment, and marital status were associated with oral health in the pooled sample. (Table 3).

### 3.4. Logistic Regressions in Non-Hispanic Whites, Hispanic Whites

Based on *Model 3*, education, income, employment, and marital status were associated with oral health in non-Hispanic Whites. Based on *Model 4*, education, income, employment, and marital status were not associated with oral health in Hispanic Whites (Table 4).

## 4. Discussion

The current study was conducted with two aims: First, to test the association between SES and self-rated oral health in overall sample. Second, to compare non-Hispanic Whites and Hispanic Whites for the effects of SES on self-rated oral health. Our first finding suggested that education, income, marital status, and employment are associated with better self-rated oral health. Our second finding showed that all these SES indicators better protect non-Hispanic Whites than Hispanic Whites.

The first finding is in line with extensive theoretical and empirical work on fundamental causes [33] and social determinant [34,35,36]. Social patterning of oral health is in line with social gradient in other health domains. Most outcomes in health are socially patterned, and oral health is not an exception to this rule. Low income, low education, and unemployment reduce health [37]. This is shown across cohorts, settings, and follow up durations [38,39,40,41,42,43].

Economic resources such as employment and income are essential for maintaining health [33]. Not only high SES reduces individuals’ exposure to risk factors of tooth decay and poor oral health [44], employment and income increase population to preventive dental care [44,45,46,47]. This is particularly true for United States where employment is the gatekeeper to insurance [48]. As dental care is expensive, poor and unemployed individuals face major difficulty for maintaining oral health [49].

The second finding is in line with previous research that has shown equal resources result in unequal gain across ethnic groups, with disproportionately larger gains for Whites than minorities [50,51,52,53,54]. Most of the literature, however, is generated from comparison of Blacks and Whites, also known as Blacks diminished return hypothesis [20]. In NSAL data, SES protected individuals against poor oral health, however, these protective effects were larger for Whites than Blacks [55]. In another study, family SES (living out of poverty) had a stronger effect on dental health care use in Whites than Black children [56].

Research, however, is mostly on Blacks than other ethnic groups. In a study, education and income result in larger health gain across domains for Whites compared to Blacks [57], patterns that are similar for young people [58], adults [59] and older adults [60]. The effects of education [59], employment [60], social contacts [61], self-efficacy [62], sense of control over life [63], neighborhood quality [64], and affect [65] on mortality all are shown to be larger for Whites than for Blacks. So, SES may not be the great equalizer of ethnic groups [66].

It seems that Blacks’ diminished return [20] is a part of a more general broad pattern on minorities diminished return [15]. This suggests that it is ethnicity and SES not ethnicity or SES that cause health disparities [67].

The results of this study contribute to our understanding of causes of health disparities. Although some of the minorities’ worse health outcomes are due to lower SES that shape their resources (Differential exposures), SES disproportionately better serves the majority group than the minority populations (Differential effects). So, SES may better help the privileged group to avoid risk and minimize subsequent negative consequences of exposures and illness [68].

The message of this paper for policy makers, public health officials, and clinicians is that they should not assume that promotion of access to socioeconomic resources will similarly enhance the health of all social groups. Risk factors, protective factors, and group membership interact on health [67]. Policy makers should be aware that demographic populations with same SES will have various levels of health. Researchers should also explore interactions between ethnicity, and SES on health outcomes [69,70]. Due to a diminished return among Blacks, it is plausible to expect wide ethnic disparities at the highest levels of SES [71]. However, it is always difficult to decompose the effects of ethnicity and SES on health [72]. Due to residual and unmeasured confounding variables and considerable overlap between race and SES, more research is needed on how ethnicity impacts health [73]. Such research should also include higher level factors such as neighborhood SES and policy.

Oral health is sensitive to health behaviors from childhood [74,75]. Ethnic minorities who are able to climb the social ladder may still have spent childhood in poverty. When they were children, the unemployment, low income, and low education of parents has already taken their toll from their oral health. Minorities are more recent to middle class, that means they are more likely to have lower SES parents. Oral health may be a lower priority for low SES families, particularly those who are struggling with economic hardships and other medical needs [49,76,77]. Oral health literacy is a function of SES and ethnicity [78,79]. All these may suggest why high SES minorities may have worse oral health than high SES Whites.

### 4.1. Limitations

Current study had several limitations that limit applicability and generalizability of the results. First and foremost, this was a cross-sectional study and the results should not be regarded as causations. Second, oral health was measured using self-rated single item measure, that has limited validity. This can become a problem if the validity of such measure if a function of ethnicity. Although self-rated oral health is linked to other measures such as dental health care utilization and dental health care need, these associations are not very strong. Third, the study did not measure any mechanism for differential effects of education and income on oral health. Furthermore, the data used for this study were collected in 2003. Although the data were old, the recent political climate in United States, which has wakened the hidden racism against minorities and immigrants, may have increased the diminished return of ethnic minorities from their SES resources. Fourth, the current data were collected via face-to-face household interviews and telephone interviews. Mode of the interview may have some impact in health surveys. Only a minority (less than 20%) of the interviews, however, were conducted via phone. Sampling bias may also be a problem as some individuals could not be contacted by phone and could only undergo the face-to face-interview. Finally, the survey used for the current analysis was from 2003. Inequalities in SES as well as oral health and may have changed since then. There is a need to replicate the inequalities and gradients in oral health that were reported here in more recent US nationally representative surveys. Despite these limitations, large sample size, and nationally representative sample were two major advantages of the current study.

### 4.2. Future Research

Research is also needed on contribution of the education system, labor market, segregation, and access to care in shaping differential effects of SES on oral health of ethnic groups. There is also a need to ascertain the most effective public and health policies that minimize health disparities due to diminished gain among ethnic minorities. As the mechanisms that cause oral health disparity are multifaceted, the required research should consider inputs from public policy, economics, health policy, psychology, anthropology, sociology, epidemiology, dentistry, and public health. Future research may focus on cultural, social, and community level factors that impact these diminished gains [80,81,82,83,84,85,86]. More research is also required in an individual level, on how coping, literacy, and individual behaviors explain the differential effects of SES on oral health of populations.

Future research also needs to explore the role of smoking, oral health care, and obesity, that are linked with periodontal disease, as potential mechanism for differential effects of SES. SES, chronic disease, and periodontal conditions are all linked, thus future needs to test the role of chronic disease in diminished oral health of high SES Latinos. Future research should go beyond self-rated oral health and test oral diseases by the intersection of race and class. Future research should also include clinical examination and more detailed oral health data. National surveys such as the National Health and Nutrition Examination Survey (NHANES) data that collect detailed information on oral health are useful source for future research. Such studies may decompose the effects of race and class in shaping burden of oral health disparities [87,88,89].

### 4.3. Conclusions

To conclude, SES is protective against poor oral health overall, however, non-Hispanic Whites gain more oral health from their SES than Hispanic Whites. Role of diminished return of SES as a contributing mechanism behind ethnic health disparities should not be overlooked.

## Figures and Tables

**Table 1:** Descriptive statistics in the pooled sample and based on ethnicity.

Characteristics	All	Non-Hispanic Whites	Hispanic Whites
	**%**	**%**	**%**
Gender			
Male	52.79	52.78	53.16
Female	47.21	47.22	46.84
Employed *			
No	27.00	26.76	34.22
Yes	73.00	73.24	65.78
Married *			
No	46.19	45.82	57.07
Yes	53.81	54.18	42.93
Poor Dental Health *			
No	90.48	90.35	94.44
Yes	9.52	9.65	5.56
	**Mean**	**Mean**	**Mean**
Age *	44.86	46.73	37.96
Education *	2.56	2.69	1.97
Household Income (USD 10,000) *	5.72	6.17	4.45

* *p* < 0.05.

**Table 2:** Spearman correlations in the pooled sample.

Characteristics	1	2	3	4	5	6	7	8
1 Ethnicity (Hispanic Whites)	1.00							
2 Gender (Men)	0.03	1.00						
3 Age	−0.14 *	−0.02	1.00					
4 Education	−0.05	0.00	−0.12 *	1.00				
5 Household Income (USD 10,000)	−0.05	0.14 *	−0.08 *	0.35 *	1.00			
6 Employment (Employed)	−0.03	0.09 *	−0.40 *	0.28 *	0.30 *	1.00		
7 Marital Status (Married)	−0.03	0.16 *	−0.06	0.02	0.38 *	0.12 *	1.00	
8 Self-Rated Oral Health (Poor/fair)	−0.03	−0.01	0.10 *	−0.17 *	−0.15 *	−0.17 *	−0.09 *	1.00

* *p* < 0.05.

**Table 3:** Summary of logistic regressions between SES (education, income, employment, and marital status) and poor self-rated mental health (SRH) in the pooled sample.

Characteristics	OR	OR	OR	OR
	Education	Income	Employment	Marital Status
**Education**				
Ethnicity (Hispanic Whites)	0.56 *	0.49 *	0.52 *	0.64 *
Gender (Men)	1.69 *	2.35 *	2.07 *	1.89 *
Age	1.02 *	1.02 *	1.01 *	1.02 *
SES	0.57 *	0.65 *	0.28 *	0.47 *
Intercept	0.15 *	0.13 *	0.12 *	0.04 *
**Income**				
Ethnicity (Hispanic Whites)	0.53 *	0.10 *	0.43 *	0.37 *
Gender (Men)	1.69 *	2.36 *	2.07 *	1.89 *
Age	1.02 *	1.02 *	1.01 *	1.02 *
SES	0.57	0.63 *	0.28 *	0.46 *
SES * Ethnicity	1.02 *	1.68 *	1.55 *	3.37 *
Intercept	0.15 *	0.14 *	0.13 *	0.04 *

OR: Odds Ratio. SES (Socioeconomic Status). * *p* < 0.05.

**Table 4:** Summary of logistic regressions between SES (education, income, employment, and marital status) and poor oral self-rated health (SRH) in non-Hispanic and Hispanic Whites.

	OR	OR	OR	OR
	Education	Income	Employment	Marital Status
***Model 3***				
**Non-Hispanic Whites**				
Gender (Women)	1.76	2.50 *	2.17 *	1.98
Age	1.01	1.02	1.00	1.02
SES	0.57 *	0.63 ***	0.27 *	0.45 *
Intercept	0.15	0.13 *	0.13	0.04 **
***Model 4***				
**Hispanic Whites**				
Gender (Women)	0.18 #	0.11 *	0.15 #	0.10 #
Age	1.04 *	1.04 **	1.04 *	1.05 ***
SES	0.71	1.14	0.76	3.17
Intercept	0.05 *	0.01 ***	0.03 *	0.01 ***

SES (Socioeconomic Status). OR: Odds Ratio. ^#^ *p* < 0.1, * *p* < 0.05, ^**^ *p* < 0.01, ^***^ *p* < 0.001.
